# Locally linear attributes of ReLU neural networks

**DOI:** 10.3389/frai.2023.1255192

**Published:** 2023-11-23

**Authors:** Ben Sattelberg, Renzo Cavalieri, Michael Kirby, Chris Peterson, Ross Beveridge

**Affiliations:** ^1^Department of Computer Science, Colorado State University, Fort Collins, CO, United States; ^2^Department of Mathematics, Colorado State University, Fort Collins, CO, United States

**Keywords:** neural networks, ReLU, linearization, linear mapping, polyhedral decomposition, Jacobian matrices

## Abstract

A ReLU neural network functions as a continuous piecewise linear map from an input space to an output space. The weights in the neural network determine a partitioning of the input space into convex polytopes, where each polytope is associated with a distinct affine mapping. The structure of this partitioning, together with the affine map attached to each polytope, can be analyzed to investigate the behavior of the associated neural network. We investigate simple problems to build intuition on how these regions act and both how they can potentially be reduced in number and how similar structures occur across different networks. To validate these intuitions, we apply them to networks trained on MNIST to demonstrate similarity between those networks and the potential for them to be reduced in complexity.

## 1 Introduction

Building a better understanding of neural network behavior is critically important. Neural networks are state-of-the-art in a variety of contexts, including facial recognition (Deng et al., 2019) and object recognition (Russakovsky et al., 2015). However, there is limited understanding of how these networks work or what they are truly doing to achieve such high performance. We present one path for building understanding and intuition by investigating the locally linear behavior of ReLU networks.

ReLU neural networks can be broken into linear region facets—the small polytopes where the network behaves as a linear function based on the activation pattern of the ReLU activation functions. These can be considered both through the underlying geometry of the polytope partitioning of the network and through the linear function associated with the network within each polytope. Prior work has been done on establishing theoretical bounds on the number of regions that it is possible for a network to have (Pascanu et al., 2013; Montufar et al., 2014; Raghu et al., 2017) and on investigating metrics involving these structures (Novak et al., 2018).

Much of the original study dealing with the linear regions of ReLU neural networks has focused on investigating expressivity and complexity. It has previously been shown that networks are universal approximators, that is, subject to certain mild constraints, and they are able to approximate any well-behaved function to within arbitrary precision as the size of the network increases (Cybenko, 1989; Hornik, 1991; Hanin and Sellke, 2017; Lu et al., 2017; Lin and Jegelka, 2018). As meaningful as these results are, they are typically not applicable to practical neural networks and do not say anything about the expressivity of a *given* neural network. To assist with determining the expressivity of networks in practice, various groups found and improved bounds on the maximum number of linear regions that feedforward fully connected ReLU neural networks can attain as functions of their width, the number of nodes in a given layer, and depth, that is, the number of layers (Pascanu et al., 2013; Montufar et al., 2014; Raghu et al., 2017). The main result of this study is that the maximum number of linear regions a network can have grows polynomially in the width and exponentially in the depth (Raghu et al., 2017). This partially explains the success of the trend in many modern neural networks to go deeper, such as ResNet (He et al., 2016).

However, empirical investigations of the number of linear regions actually achieved by many neural networks have shown different results. Untrained neural networks after initialization have a number of linear regions that tends to grow linearly in the number of ReLU functions along any one-dimensional subspace of the input space (Hanin and Rolnick, 2019a). Furthermore, after training, the number of regions tends to grow polynomially in the number of ReLU nodes in the network and exponentially in the dimension of the inputs to the network (Hanin and Rolnick, 2019b).

These linear regions have also been used empirically to measure the sensitivity of neural networks. As will be discussed in Section 2.1, the Jacobian of a neural network at a point, together with the value of the neural network at the point, describes exactly the linear function that agrees with the network in a polytope around that point. Novak et al. (2018) utilized this fact to investigate the effect of hyperparameters on input sensitivity and found that overparameterization can help in generalization. Additionally, they Zhang and Wu (2019) investigated how the linear region structure can be used to predict the quality of a network.

Many of these studies have used visualization methods for the polytope structures of neural networks (Hanin and Sellke, 2017; Hanin and Rolnick, 2019a; Zhang and Wu, 2019). These visualizations are frequently done on MNIST or similar datasets using cross-sections of the input space to better understand how the polytope structure of networks evolves through training or through different training methodologies. We apply such visualizations to toy, two-dimensional input problems so that we can build intuition on problems where the entire relevant input space is viewable.

Liu et al. (2023) investigated the properties of the activation patterns of the ReLU functions as bit strings corresponding to these linear regions, although their method works only for fully connected networks, and they did not extend it to convolutional layers or max-pooling.

In the study by McNeely-White et al. (2019), it was shown that one can apply a linear map to the feature vector (the outputs of the pre-classification layer) of one network to obtain a vector, considered as a feature vector in the second network, that can then be used by the second network for classification while maintaining high accuracy.

Zhang et al. (2018) showed that due to the piecewise linear structure of these neural networks, and under certain assumptions, the set of ReLU neural networks, the set of piecewise linear functions, and the set of tropical rational functions are equivalent. We do not extend our results to the realm of tropical algebra, but we do take inspiration from the concept of the dual as commonly expressed in tropical algebra.

We investigate the behavior of linear region for small networks trained on toy problems where full visualization is possible to build intuition for the behavior and structure of both the polytope geometry and their associated linear functions. Insights from those small networks are extended to larger, more modern networks trained to recognize handwritten digits from the Modified National Institute of Standards and Technology database for handwritten digit recognition (MNIST) (LeCun et al., 1998; Szegedy et al., 2016; Lin and Jegelka, 2018). The first is that clustering these linear regions based on Euclidean distance between the weights of their linear functions can be carried out while preserving much of the original performance of the networks. This implies that networks have significant redundancy at the facet level, aligning with the success of methods for pruning and compressing networks (Frankle and Carbin, 2018; Blalock et al., 2020).

The second main result is that the linear functions associated with linear region of two different networks, trained or fine-tuned on the same problem, can be related by a linear map that maintains high accuracy. This implies that qualitatively different networks result in similar solutions when considered on the polytope level, while also providing a way to identify when two networks may identify different patterns in the input data that they exploit for classification. Identifying when networks converge to similar solutions allows for a stronger ability to determine where different architectures or training methods will be successful.

## 2 Materials and methods

Neural networks with piecewise linear activation functions, such as ReLU, are continuous piecewise linear maps from the input space to the output space (Zhang et al., 2018). Additionally, each of the linear portions of this mapping is supported on a convex polytope defined by the boundaries along which the ReLU nodes activate. Visualizing and analyzing the structure of these linear regions allows for increased understanding of network behavior.

### 2.1 Linear regions definition

The piecewise linear and convex polytope structures of a ReLU neural network, *f*:ℝ^*d*^ → ℝ^*o*^ with inputs in ℝ^*d*^ and *o* outputs, mean that it can be written as a piecewise linear function (Zhang et al., 2018). A representation of that is


(1) $$f(x)=\{\begin{array}{ll} W_{1}x+b_{1}, & \text{if }A_{1}x\leq c_{1}\\ W_{2}x+b_{2}, & \text{if }A_{2}x\leq c_{2}\\ \vdots & \;\\ W_{m}x+b_{m}, & \text{if }A_{m}x\leq c_{m}. \end{array}$$

**Figure 1:**
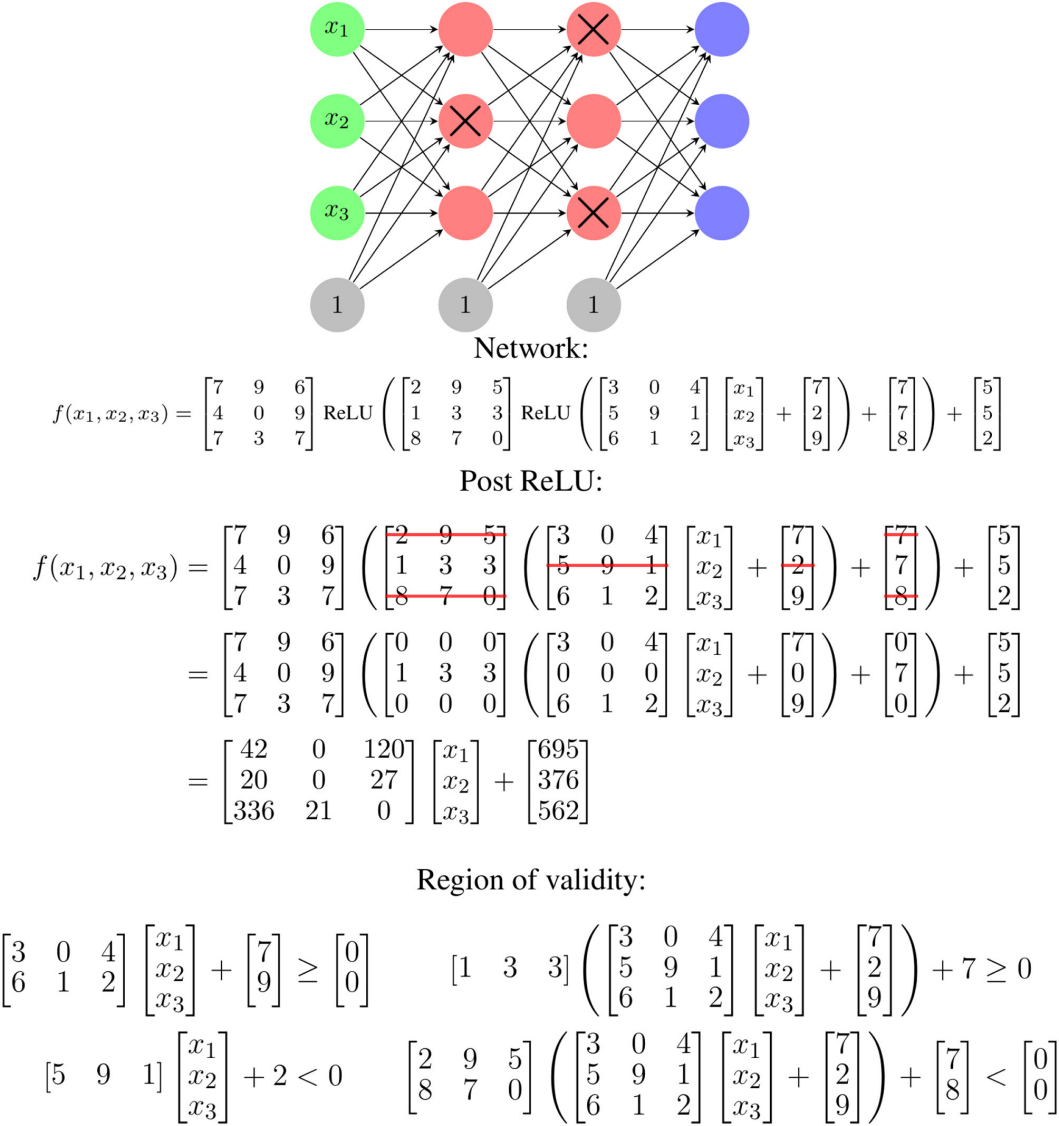
An illustration of how the ReLU activation pattern for an input determines the linear mapping used for that input. If a given input fails to activate the crossed out red nodes, the corresponding rows of matrices in the functional representation are zeroed. This leads to a single linear function of the input for that input. The region of validity refers to the possible *x* values for which this ReLU activation pattern exists and is determined by finding inequalities corresponding to the zeros of the ReLU function. The zero for a given node corresponds to the *x* values for which the output value of the associated matrix row is zero. All the equations must be satisfied.

For each of the 1 ≤ *i* ≤ *m* linear regions, the affine mapping defined by **W**_*i*_ and *b*_*i*_ is valid on the convex polytope defined by **A**_*i*_ and *c*_*i*_. One way to determine these parameters for a given input, *x*, starts with identifying which ReLU functions are activated for that input. Zeroing the weights in the network associated with deactivated ReLU nodes and converting activated ReLU functions to the identity function, **W**_*i*_ and *b*_*i*_ can be determined by multiplying through the resulting linear equation. The values of **A**_*i*_ and *b*_*i*_ can be determined by finding the zeros of the ReLU functions and setting inequalities based on their activation patterns. An example of this process is shown in Figure 1. This piecewise linear mapping structure can be extended to various other common layers types, such as max and average pooling with additional work.

The **W**_*i*_ and **A**_*i*_ are linked—the **W**_*i*_ are selected based on which ReLU nodes are activated, and the **A**_*i*_ describe where ReLU nodes switch from activated to deactivated or vice-versa. This is partially illustrated in Figure 1 and a specific, smaller example of this is shown later in Equations 2 and 3. There are also similarities and relationships between different **W**_*i*_ or **A**_*i*_—because they are coming from the same network weight matrices with rows removed, there is an inherent structure in the specific values used to construct them.

An additional note to make is that the number of regions, *m*, has the potential to be very large, with exponential growth in the depth and polynomial growth in the width of the network (Pascanu et al., 2013; Montufar et al., 2014; Raghu et al., 2017). Experimentally, trained networks have been shown to typically exhibit polynomial growth with the number of ReLU activations of the network, where the degree of the polynomial is the input dimension (Hanin and Rolnick, 2019b). Although this is polynomial, networks applied in domains such as image recognition frequently have inputs with at least 1,000 dimensions, so this still results in very large numbers of regions (Russakovsky et al., 2015).

The linear mapping network definition, Equation 1, highlights the fact that as long as one of the ReLU nodes does not switch from “activated” to “deactivated” or vice-versa, the behavior of the network is purely linear. Since the network is a composition of continuous linear and piecewise linear functions, it is itself a continuous piecewise linear function that splits the input space into disjoint polytopes, on each of which there is an associated affine mapping. This represents an unequivocally simple way to conceptualize what ReLU networks compute, but unfortunately, the typically extreme growth in the number of facets in Equation 1 means enumerating the full set of affine mappings is impractical for most modern networks.

Because of this difficulty in computation, Equation 1 is of conceptual value but, arguably by itself, not of much practical value; however, it leads to several distinct yet ultimately equivalent views of neural networks. Some of the relevant views are:

- The weight matrix, **W**_*i*_, is the Jacobian of the neural network in the region described by **A**_*i*_ and *c*_*i*_. The *j*^th^ row of **W**_*i*_ is the gradient of the *j*^th^ output of the network. This fact has been utilized previously to consider sensitivity metrics for neural networks (Novak et al., 2018). This also allows for simple calculation of the **W**_*i*_ and *b*_*i*_ values, even in networks with unusual piecewise linear activation functions.
- The weight matrices, **W**_*i*_, and biases, *b*_*i*_, form a set of linear maps which the neural network chooses from based on the value of the input. Each row of these **W**_*i*_ is a surface normal to the hyperplane used for classification.
- The choices are based on the location of the input in a set of connected polytopes induced by the ReLU structure of the network. We provide animations showing how these structures evolve as networks train in Section 3.1.
- Each row of **W**_*i*_ concatenated with the corresponding element of *b*_*i*_ forms a point in ℝ^*d*+1^. These points can be considered as lying in a “dual” space to the corresponding output of the network, and their structure can be analyzed in that context to investigate the linear function behavior. We show how this space forms in Sections 2.2 and 3.1, and analyze this space for clustering and similarity of networks in Sections 3.2 and 3.3.

### 2.2 Example on XOR

**Figure 2:**
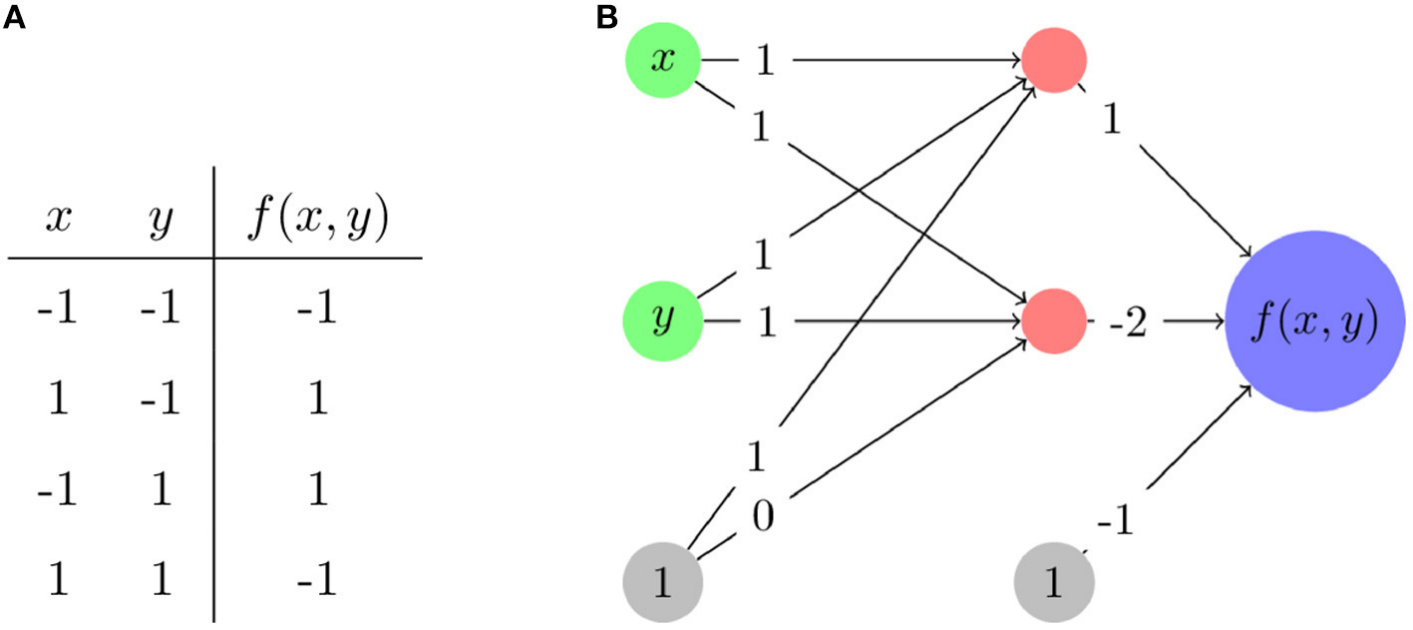
The modified XOR problem. **(A)** The input and output values—inputs and outputs are rescaled to be from –1 to 1 rather than from 0 to 1. **(B)** A network architecture and its associated weights that solves this problem. Nodes in red have ReLU applied after calculating their associated input values.

For an example of how the piecewise linear nature of ReLU neural networks works, we consider an XOR problem and a ReLU neural network that solves it as presented in Figure 2. We choose XOR as it is a complex enough problem that it illustrates non-linear aspects of network behavior, but simple enough that full analysis of that behavior is feasible. Note that for the XOR function itself, shown in Figure 2A, zero is replaced with −1 to assist with training of networks. Figure 2B shows a network which solves the XOR problem. The functional form of that same network mapping from the two inputs *x* and *y* may be written as


(2) $$\begin{array}{l} f\left(x,y\right)=\left[\begin{array}{cc} 2 & -4 \end{array}\right]\max\left\{\left[\begin{array}{cc} 1 & 1\\ 1 & 1 \end{array}\right]\left[\begin{array}{c} x\\ y \end{array}\right]+\left[\begin{array}{c} 1\\ 0 \end{array}\right],\left[\begin{array}{c} 0\\ 0 \end{array}\right]\right\}-1. \end{array}$$

As a function on ℝ^2^, the network divides ℝ^2^ into three linear regions with corresponding linear function/polytope pairs,


(3) $$f(x)=\{\begin{array}{ll} -2x-2y+1, & \text{​​​}0\leq x+y,\text{ Both ReLUs activated}\\ 2x+2y+1, & \text{​​​​​}-1\leq x+y\leq 0,\text{ Top activated; bottom}\\ \; & \text{​​​​​ not activated}\\ -1, & \text{​​​​​}x+y\leq -1,\text{ Neither ReLU activated} \end{array}$$

**Figure 3:**
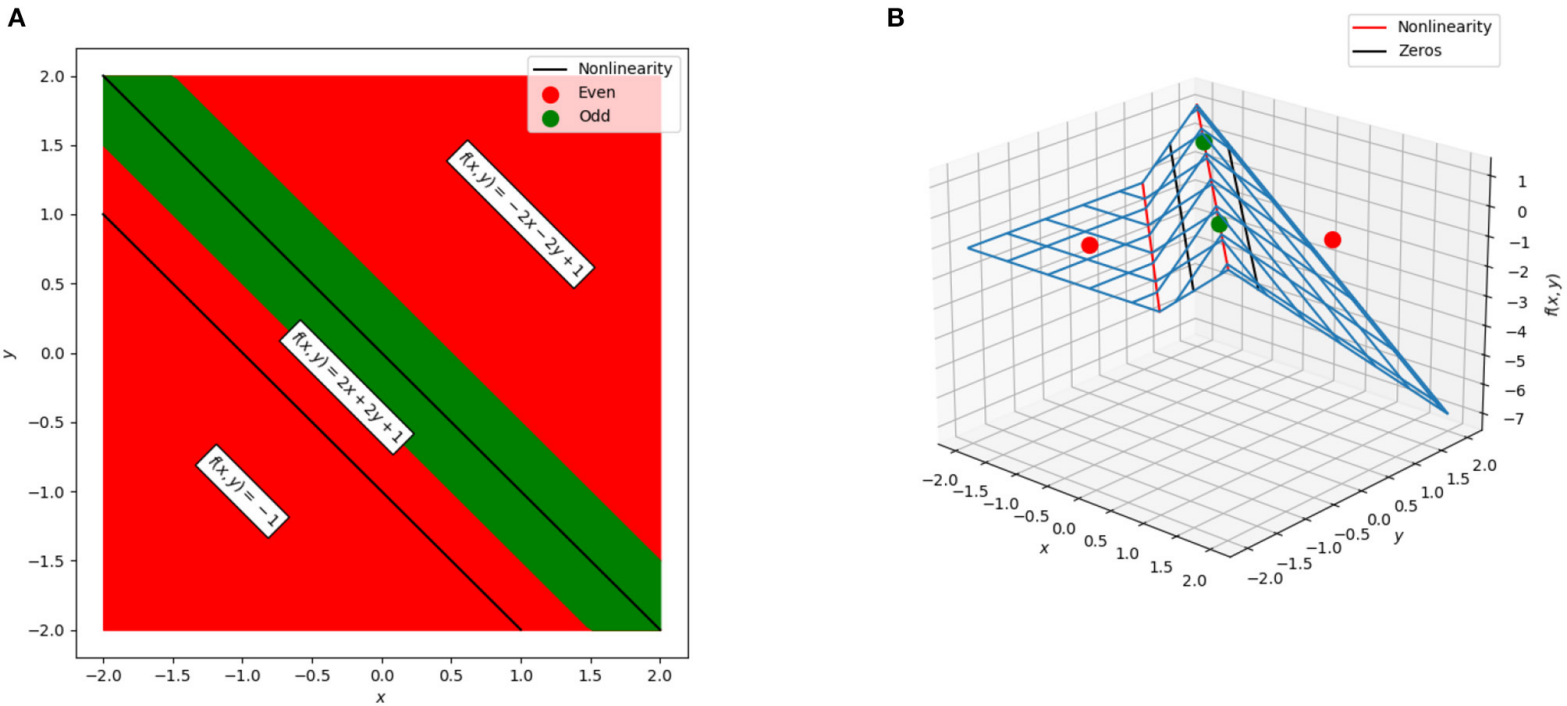
The polytopes and associated linear regions for a simple network to solve the XOR problem. **(A)** The cross-section of the network in the plane. Green corresponds to points that would be labeled in the positive class (neural network output greater than zero) and red corresponds to points that would be labeled in the negative class (neural network output less than zero). The black lines correspond to the points at which one of the two ReLU units “activates” or “deactivates” and switches the linear region used for classification. The three polytopes form bands in the plane. **(B)** The surface of the neural network. The points used for training are shown as green and red dots, the non-linearities are shown as red lines, and the decision boundary (zeros of the network) are shown as black lines.

These linear regions are shown in Figure 3. Even for this very simple example, a complication arises: there is actually a “fourth” region, −4*x*−4*y*−1, tied to the case where the lower ReLU node is activated and the top is not. However, that case occurs in the empty polytope 0 ≤ *x*+*y* ≤ −1 which cannot occur for any values of *x* and *y*, and thus, in practical terms, this empty polytope does not exist. This is an example of a general phenomena where cases exist in principle but are unreachable regardless of input. Furthermore, the existence of such cases explains in part why the number of possible linear regions grows as it does and not simply as a power of two of the number of ReLU functions.

There are additional practical complications that can arise but do not on this network due to its simplicity—a network can be considered as a function on all of its input space, ℝ^*d*^, but the data to which the network is actually applied lie in a bounded region within that space. Polytopes may exist outside of that region but not be meaningful for the purpose of the network. Furthermore, in many problems, the data used are a discrete subset of this bounded region. It is possible for the network to define polytopes lying in the bounded region but too small to contain any of the discrete data to which the network is applied. In general, we observe that the number of polytopes does typically grow beyond the number of actual training samples when considering high-dimensional input data and complex networks.

**Figure 4:**
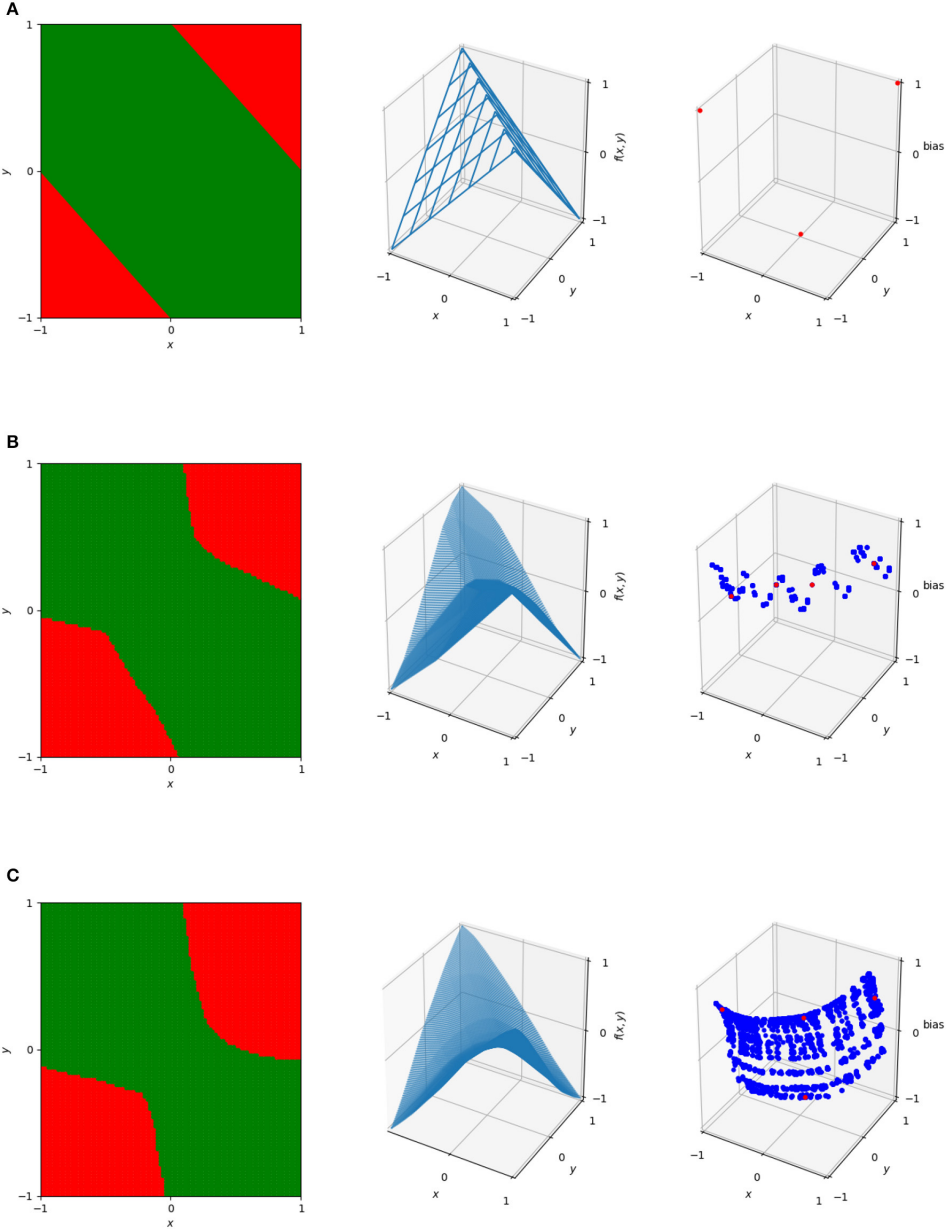
The decision boundaries (left), wireframe representations of output (center), and dual representations of the linear regions (right) for three networks designed to solve ReLU. The top network **(A)** is the simple one described previously. The center **(B)** and bottom **(C)** are single hidden layer neural networks with the center having 20 hidden nodes and the bottom having 100 hidden nodes. In the dual, blue dots represent linear regions used on the 101 × 101 uniform grid in [−1, 1]^2^. The red dots represent the linear regions used for the actual classification of the four data points—note that the top image only has three dots corresponding to these, rather than four, as it only has a total of three linear regions.

Returning to the regions shown in Figure 3, the weights and biases in these polytopes can be considered as *d*+1-dimensional points existing in a “dual space” to the original neural network. For example,


(4) $$\begin{array}{l} -x-y=\left[\begin{array}{ccc} -1 & -1 & 0 \end{array}\right]\left[\begin{array}{c} x\\ y\\ 1 \end{array}\right] \end{array}$$

and so the point (−1, −1, 0) in the dual is induced by this region. Further examples of these duals are illustrated in Figure 4, which shows the decision boundary, numerical output, and dual points for three networks with varying numbers of nodes trained on the XOR problem. These can illustrate patterns in the behavior of the network, and as will be discussed in more detail, mapping between dual regions of networks or clustering in this space can identify similarity metrics and areas where the neural network gives potentially unnecessary complexity.

### 2.3 Polytope visualization

**Figure 5:**
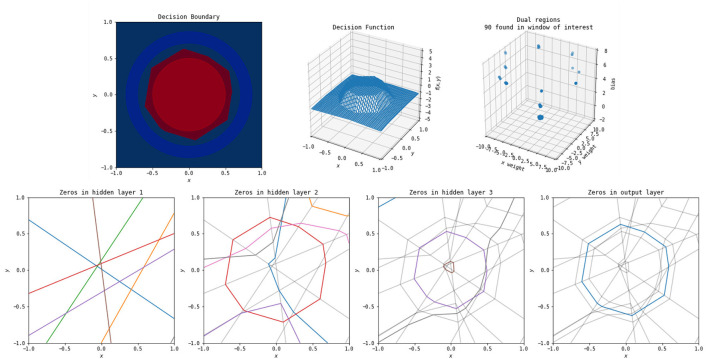
The polytopes resulting from the various layers of a simple network to classify a circle versus a surrounding annulus. **Top left**: the original problem and the decision boundary determined by the network. **Top middle**: the outputs of the network. **Top right**: the dual weights. **Bottom first**: the zeros for each of the perceptrons from the original input space to the first hidden layer—these decision boundaries are all lines, as the perceptrons at this stage are purely linear in the original input. Each color corresponds to one of eight nodes in this hidden layer (and the colors do not relate between each of the four bottom plots). Not every node has zeros occurring in the window shown. **Bottom second**: the zeros for each of the perceptrons from the original input to the second hidden layer, with the boundaries for the first hidden layer in light gray. These are lines in the output space of the first layer, but appear non-linear when shown in the original input space. Each boundary can only break at one of the lines from the previous layer. **Bottom third**: the zeros for each of the perceptrons from the original input to the third layer, with the boundaries of the first two layers in gray. Breaks in this layer can occur at any location where it crosses a zero of a previous layer. **Bottom fourth**: zeros in the output layer. This forms the decision boundary shown in the top left.

One way to think of the polytopes resulting from ReLU activation patterns is the way in which they arise as a consequence of the iterated perceptron structure inherent in this style of network. Each ReLU node in the network builds upon the non-linearities in the previous layers by having its activation boundary correspond to a line in the output space of the previous layer. An example of this is illustrated in Figure 5. This figure shows the decision surface, numerical output, dual points, and the boundaries of the linear regions induced by each node split by layer.

The boundaries induced by the first hidden layer of the network, bottom left of Figure 5, are relatively simple—each of the nodes in the first layer has a line in the original input space for an activation boundary where the output of that node switches from positive to negative. Each subsequent layer builds upon the previous. However, the activation boundaries of ReLU nodes in subsequent layers form lines in *compressed* space. Within the polytopes formed by the activation boundaries of previous layers, the new activation boundaries are still linear, but when changing between those polytopes, the new activation boundaries are able to change angle. To illustrate this, the activation boundaries for each layer in Figure 5 are reproduced in subsequent layers in gray. The more complicated activation boundaries for each subsequent layer are always locally linear with changes in direction only arising where they intersect a boundary from a previous layer. This is a direct result and also illustration of the fact that the non-linearities of multi-layer ReLU networks must be built up from activation boundaries established by the previous layers in the network. Finally, notice in the bottom right of Figure 5 that the output layer of the network does as expected, constructing a valid piecewise linear decision boundary for the original classification task.

A few things can be noticed from Figure 5. The first is that the final decision boundary is not reliant on all of the activation boundaries from previous layers. This implies that some of the nodes in the network could be removed without qualitatively impacting the classification. Additionally, the final decision boundary corresponds closely to an activation boundary in the second hidden layer, suggesting that layers after the second are not necessary. These observations support the idea of the “lottery hypothesis,” where networks have more nodes that necessary, and subnetworks that are initialized well can be the main driving force for network success (Frankle and Carbin, 2018; Blalock et al., 2020). Finally, the 90 identified linear function weights in the dual graph cluster into a small number of points, again suggesting simplification of the network is possible to remove such redundancy.

### 2.4 Region modification

To investigate extraneous complexity in networks, it is useful to consider the linear functions that arise on each polytopes. This is useful for a number of reasons, but the two simplest are that the visualizations done in the previous section cannot be done as simply with high-dimension input data, and that the complexity of the polytope partitioning increases significantly with the complexity of the network. Using the linear functions allows us to sample points from the input space and compare the behavior of a network or networks across those points without having to worry about the polytope structure between those points. Even for relatively simple image classification datasets such as MNIST, small networks have nearly every image in the dataset lying on a unique polytope (Novak et al., 2018). Additionally, by calculating the linear functions using the Jacobian, we can largely treat the network structure as a black box and avoid difficulties that arise from considering more complex activation functions (Liu et al., 2023).

For investigating these affine mappings, there are two useful steps to take: constructing notation to allow us to refer to the set of affine mappings potentially used for a specific output of a network, and considering only the affine mappings that are used for training or testing to reduce the number to something computationally manageable.

In terms of notation, the **W**_*i*_, which is the Jacobian of the network within the region defined by **A**_*i*_ and *c*_*i*_, and *b*_*i*_ described in Equation 1 can be written as


(5) $$\begin{array}{l} {\text{W}}_{i}=\left[\begin{array}{c} w_{i,1}^{T}\\ w_{i,2}^{T}\\ \vdots \\ w_{i,o}^{T} \end{array}\right]\text{and}b_{i}=\left[\begin{array}{c} b_{i,1}\\ b_{i,2}\\ \vdots \\ b_{i,o} \end{array}\right], \end{array}$$

Where each *w*_*i, j*_ and *b*_*i, j*_ correspond to the affine mapping in polytope 1 ≤ *i* ≤ *m* for the 1 ≤ *j*^th^ ≤ *o* output of the network. Then, it is possible to construct the matrix containing the set of affine mappings used for a given output, *j*, as


(6) $$\begin{array}{l} {\bar{\text{C}}}_{j}=\left[\begin{array}{cc} w_{1,j}^{T} & b_{1,j}\\ w_{2,j}^{T} & b_{2,j}\\ \vdots \\ w_{m,j}^{T} & b_{m,j} \end{array}\right]\in {\mathbb{R}}^{m\times \left(d+1\right)}. \end{array}$$

In practice, it is computationally infeasible to calculate all *m* linear regions, so for the purpose of empirical studies, we choose *p* points in the input space to sample and construct the matrix.


(7) $$\begin{array}{l} {\text{C}}_{j}=\left[\begin{array}{cc} w_{1,j}^{T} & b_{1,j}\\ w_{2,j}^{T} & b_{2,j}\\ \vdots \\ w_{p,j}^{T} & b_{p,j} \end{array}\right]\in {\mathbb{R}}^{p\times \left(d+1\right)}. \end{array}$$

For simple, two-dimensional input problems, we choose the *p* points by sampling from a uniform grid. We also consider the MNIST dataset (LeCun et al., 1998), where the *p* points we sample from are the 60,000 training or 10,000 testing input samples from that network. We construct the **C**_*j*_ matrices using the training samples, and we additionally construct ${\tilde{\text{C}}}_{j}$ using the testing samples for evaluation of how various modifications impact accuracy on the testing set.

#### 2.4.1 Clustering regions

Even for potentially large numbers of sampled affine maps, it is likely that many samples will have a unique **W**_*i*_ due to the large number of total linear regions. For example, even simple networks on the MNIST dataset only have overlap on < 1% of the training inputs. This is not necessarily surprising, simply due to the sheer number of possible linear regions the network can construct.

However, although these weights are not necessarily equivalent, there is potentially a great deal of redundancy or similarity among them. As discussed in Section 2.3 and shown in the behavior of the dual points of increasingly complex networks in Figure 4, patterns appear in the linear weights that can indicate redundant behavior. We can cluster these linear weights and determine how well those clusters are able to replicate the behavior of the network as a measure of that redundancy.

1. Calculate the **C**_*j*_ and ${\tilde{\text{C}}}_{j}$ matrices.
2. Train *k*-means clustering models using the rows of each of the **C**_*j*_ matrices.
3. For each row of each of the ${\tilde{\text{C}}}_{j}$ determine for which cluster center it is closest.
4. Use that cluster center as a linear mapping from input space to determine the value for that output.
5. Classify the input based on which of the newly calculated outputs is highest.

By varying *k* and comparing the resulting accuracy against the original accuracy of the network, we can investigate the degree to which networks can be simplified. If applying this method with *k* = 1 results in near-original accuracy, that suggests the network is behaving holistically as a linear mapping, whereas if it results in near-random accuracy, that suggests the network's behavior can not be well described by a linear transformation from the input space to the output space. Determining at which value of *k* accuracy approaches the original provides a way to understand how significantly the network can be simplified.

#### 2.4.2 Affine maps between linear functions

Another area where representing the weights of these linear regions as points in space can be useful is in finding similarities between two networks. Given **C**_*j, network*1_ and **C**_*j, network*2_, we can train least-squares regression models for each output to find matrices ${\text{M}}_{j}\in {\mathbb{R}}^{d+1\times d+1}$ for each 1 ≤ *j* ≤ *o* that minimize


(8) $$\begin{array}{l} ||{\text{C}}_{j,network1}{\text{M}}_{j}-{\text{C}}_{j,network2}|{|}_{2}. \end{array}$$

This method finds a mapping between the linear region weights, or equivalently, between the gradients of the outputs with respect to the input. Due to this, as with the *k*-means clustering method, this method requires running inputs through each original network, calculating the Jacobians, then applying the transformation.

This is similar to the study by McNeely-White et al. (2019) where the authors demonstrated that the outputs of the final layer before the linear classifier of networks trained on ImageNet are affine-equivalent. Unlike their study, our study investigates the connection between the affine mappings of the locally linear functions of networks, rather than the feature vectors of networks.

**Figure 6:**
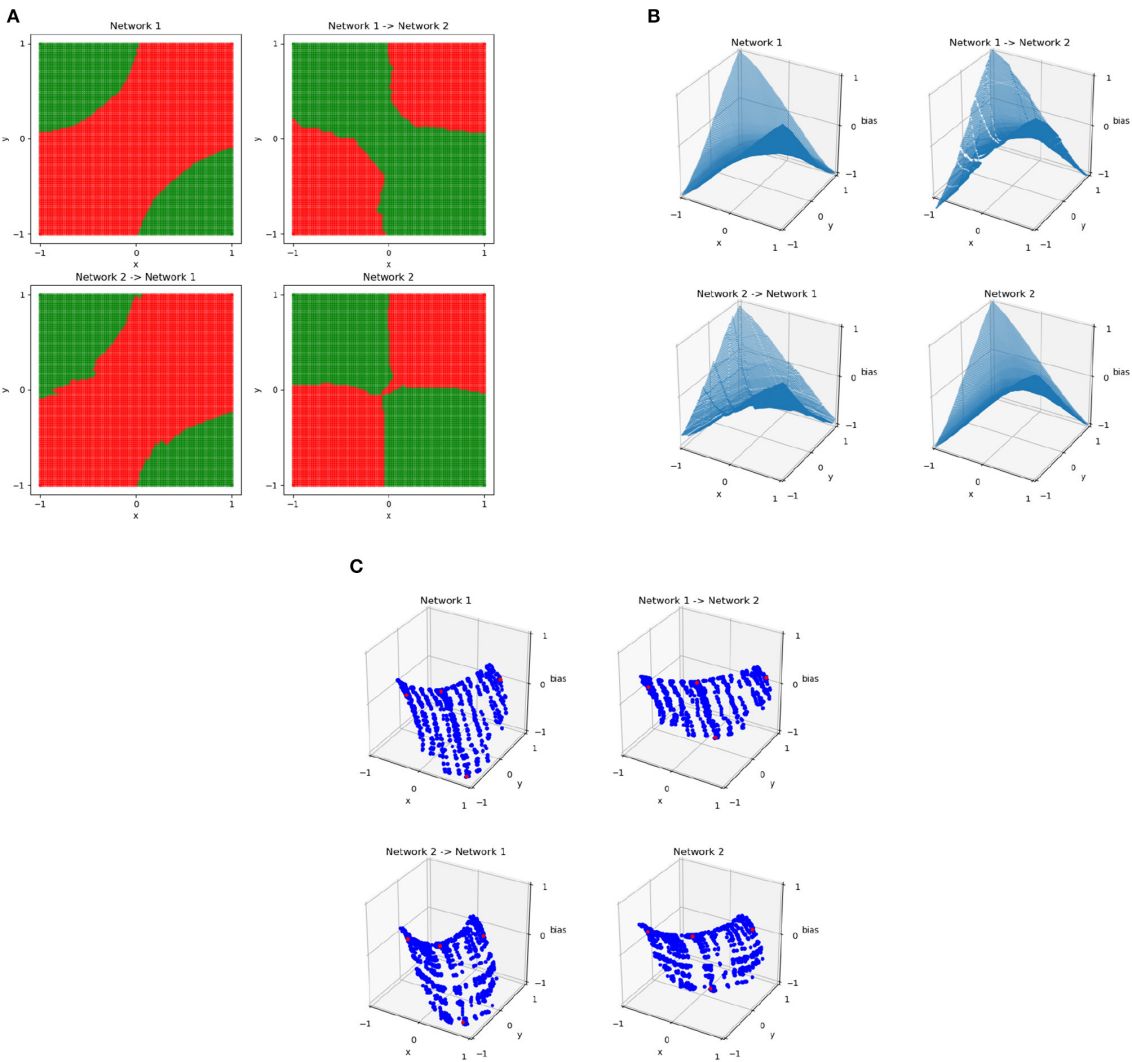
The decision boundaries **(A)**, wireframes **(B)**, and weights in the dual space **(C)** for two different XOR networks and the result of training an affine mapping from the linear regions of one network to the other. The original networks are the upper left and lower right in each subplot, with the resulting mapped networks are the top right and bottom left. In the dual space, the four points of XOR are in red and all other points sampled uniformly from the grid in the original input space are in blue.

Results of this process for XOR networks using the **C** matrices constructed by sampling points on the 101 × 101 uniform grid are shown in Figure 6, which shows the results of mapping between two networks trained on XOR. Although the two networks have similar behavior, their decision boundaries are somewhat different and their dual representations are close to rotations of each other. The resulting points of **C**_*j, network*1_**M**_*j*_ are very similar to **C**_*j, network*2_ and vice versa, meaning that the mapping is successful. The function resulting from this is no longer continuous—because the bias is part of what is being mapped, the result is able to vary based on the position in the plane and regions may no longer join at their boundaries. By applying this method to more complex networks where similarity is not as clear, we can determine potential overlap in network behavior.

### 2.5 Extension to image data

The idea of investigating and visualizing linear regions can be extended to higher dimensions, and specifically to image data, although visualizations are no longer as simple. We consider the MNIST dataset of handwritten digits which contains 60,000 training samples and 10,000 test samples (LeCun et al., 1998). MNIST was chosen as an image classification dataset due to its relative simplicity. We used PyTorch (Paszke et al., 2019) to train four networks on the MNIST dataset. These networks are

- A fully connected feedforward network with a single hidden layer consisting of 128 ReLU nodes. This network achieves an accuracy of 96.03%. The training process used cross-entropy loss and PyTorch's SGD function with parameters of 0.01 update rate, 0.5 momentum, 0.01 weight decay, and a batch size of 64 over 30 epochs.
- Two simple convolutional networks with equivalent architectures but different initializations. A convolutional layer with 10 filters and a kernel size of 5 is applied to the input, followed by a max pool. The results of that have a convolutional layer with 20 filters and a kernel size of 5 applied, again followed by a max pool. The 320 resulting outputs are used as inputs to a fully connected layer with 50 outputs, which is followed by a linear layer from those 50 nodes to the 10 outputs. The network achieves an accuracy of 98.07% (labeled Conv1) and 98.04% (labeled Conv2). The training process for both used cross-entropy loss and PyTorch's SGD function with parameters of 0.01 update rate, 0.5 momentum, 0.01 weight decay, and a batch size of 64 over 30 epochs.
- A network with the Inception-v3 architecture as implemented in Torchvision's models subpackage trained from scratch (Marcel and Rodriguez, 2010; Szegedy et al., 2016). This network achieves an accuracy of 99.08%. The first layer of the network was modified to expect images with only one channel and images were upsampled, using bilinear interpolation, to the expected size of 224 × 224 pixels. The training process used cross-entropy loss and PyTorch's SGD function with parameters of 0.1 update rate, 0.9 momentum, 1e-4 weight decay, and a batch size of 50 over 22 epochs.
- A network with the ResNet-152 architecture as implemented in Torchvision's models subpackage trained from scratch (Marcel and Rodriguez, 2010; Lin and Jegelka, 2018). This network achieves an accuracy of 98.92%. The first layer of the network was modified to expect images with only one channel. The training process used cross-entropy loss and PyTorch's SGD function with parameters of 0.1 update rate, 0.9 momentum, 1e-4 weight decay, and a batch size of 50 with 60 epochs.

**Figure 7:**
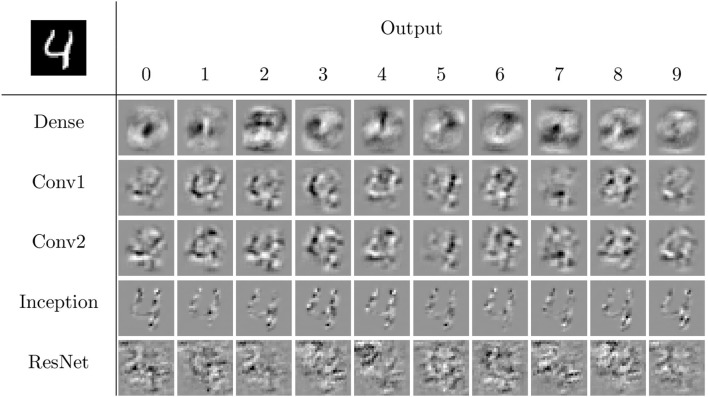
The linear regions for each output of the four networks for the input in the top left corner. These visualizations are similar to simple forms of saliency mapping (Simonyan et al., 2013). White pixels correspond to positive values, black pixels to negative values, and gray pixels to zero.

For a given input image and output, each network determines a polytope within the input space which contains the image. By considering a single output, the gradient at the input image can be displayed in the same format as the input image. The collection of 50 different gradient “images,” computed by considering each of the five neural networks and each of the 10 output nodes for an example “4” image, is visualized in Figure 7. Based on the appearance of the images, the dense network appears to have relatively little complexity, so it is classifying based on its “ideal” shape of each output, corresponding to what a linear classifier may do. This suggests a possibility for a reduction of number of linear regions used, as presented in Section 2.4.1 and discussed in Secton 3.2. The other networks have more complexity, tend to focus more sharply on the relevant information being passed in, and classify based on that input. ResNet has behavior that is not human-interpretable and appears somewhat noisy. Based on these visuals, the only networks that appear visually similar are the two simple convolutional networks, suggesting possible difficulty in the mappings presented in Section 2.4.2 and discussed in Section 3.3. The visualization of these linear regions is similar to the idea of saliency mappings, although many modern forms of saliency mapping are more sophisticated than simply visualizing the gradients at an input image, as this is doing (Simonyan et al., 2013).

## 3 Results

Constructing animations and visualizations of how network structure changes throughout the training process for different problems and architectures on two-dimensional problems can aid in understanding how the training process creates some of the properties investigated and provide inspiration for behavior to investigate in more detail. Although two-dimensional problems are useful for building intuition of network behavior, they do not necessarily include all of the behavior that most modern neural networks include. As such, using intuitions gained on those networks, even on a simple dataset such as MNIST, is necessary for confirming that networks can be reduced in complexity or exhibit quantitatively similar behavior despite differences in architecture.

### 3.1 Polytope evolution through training

**Figure 8:**
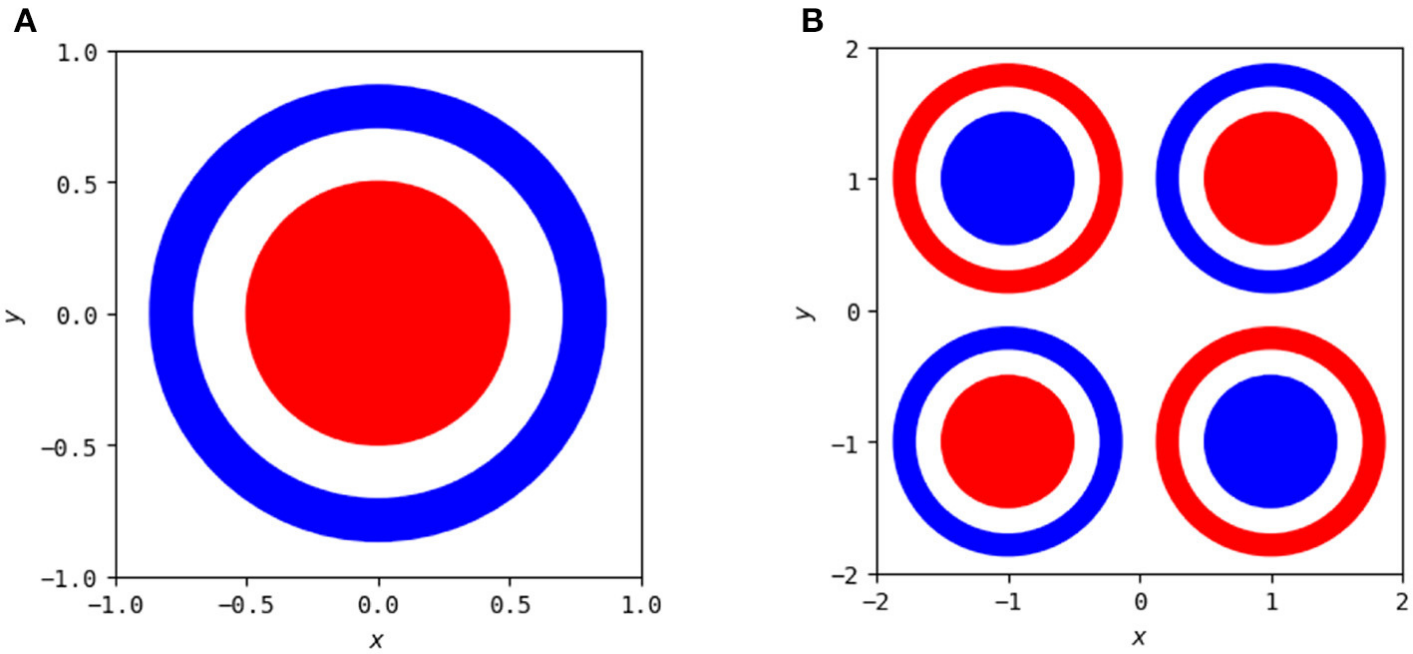
Two classification problems used to show animations of polytope structures during the training process. **(A)** The goal is to classify points in the red annulus as being a separate class as those in the blue annulus. **(B)** A combination of the left problem with the XOR problem to demonstrate more sophisticated network behavior.

The polytope structures discussed in Section 2.3 and their associated linear mappings change as the network trains, as has been studied previously (Hanin and Sellke, 2017; Hanin and Rolnick, 2019a; Zhang and Wu, 2019). However, these studies focus on MNIST and the usage of summary statistics to analyze behavior beyond the visuals in high-dimensional space. We focus on the visualization for two-dimensional input problems here, so that we can fully visualize the polytope structure and identify patterns of behavior across the entirety of the input. We continue with the problem of classifying a circle versus a surrounding annulus and additionally consider a more complex problem that is a combination of the XOR problem and the circle versus annulus problem, both illustrated in Figure 8.

There exist many simple solutions to the single circle versus single annulus problem, but neural networks do not intrinsically take advantage of the rotational symmetry of this problem to express these solutions. As has been demonstrated previously (Hanin and Sellke, 2017; Raghu et al., 2017), any network that solves this problem requires a minimum of three hidden nodes in at least one of its hidden layers. A node in any layer creates a non-linearity along a line in the input space of that layer, but when mapped back to the original input space that line becomes a trajectory that “breaks” by changing direction whenever it encounters a line created by the activation boundary of a node in a previous layer. A network with a maximum width of two is unable to solve this problem as it is unable to create a closed region in the input space. To see this, note that each layer with at most two nodes can only partition space into four regions (both on, one on, the other on, and both off), one of which (both off) will be constant. Due to this, any such network cannot form a closed region in space and will instead have each of its polytopes extend to infinity.

**Figure 9:**
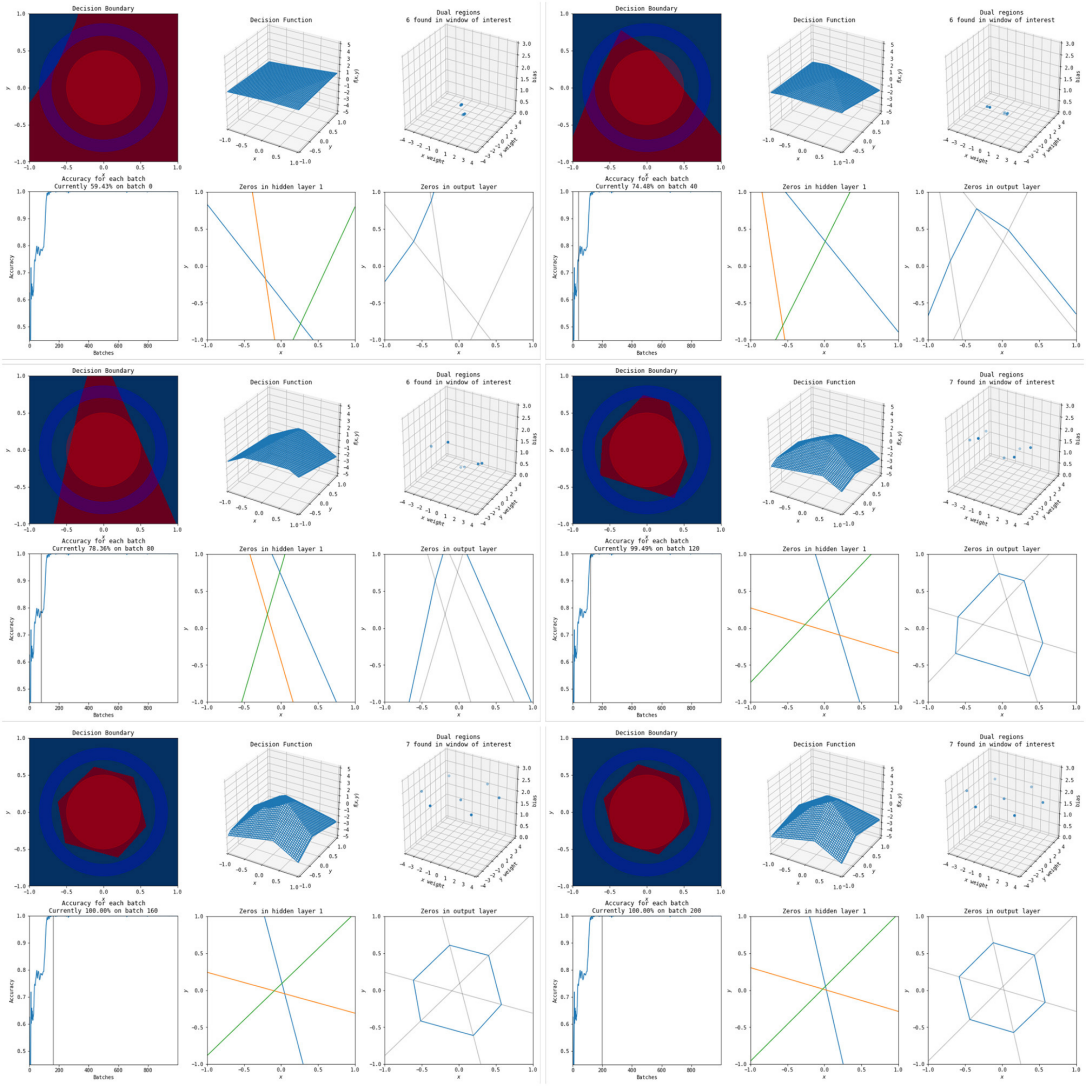
A selection of images throughout the training process of the simplest possible network (three hidden nodes in a single layer) on this problem. The **top left** set of six image shows the network at initialization, and each set of six images following that increases by 40 training epochs. Within each set of six images, the **top left** shows the decision surface, the **top center** shows the network output, the **top right** shows the weights in the dual, the **bottom left** shows the training accuracies, the **bottom middle** shows the activation boundaries of the hidden nodes, and the **bottom right** shows the decision boundary overlaid with the previous layers activation boundaries. An animation of this process is available at https://www.youtube.com/watch?v=lpXQI-UJIZM.

**Figure 10:**
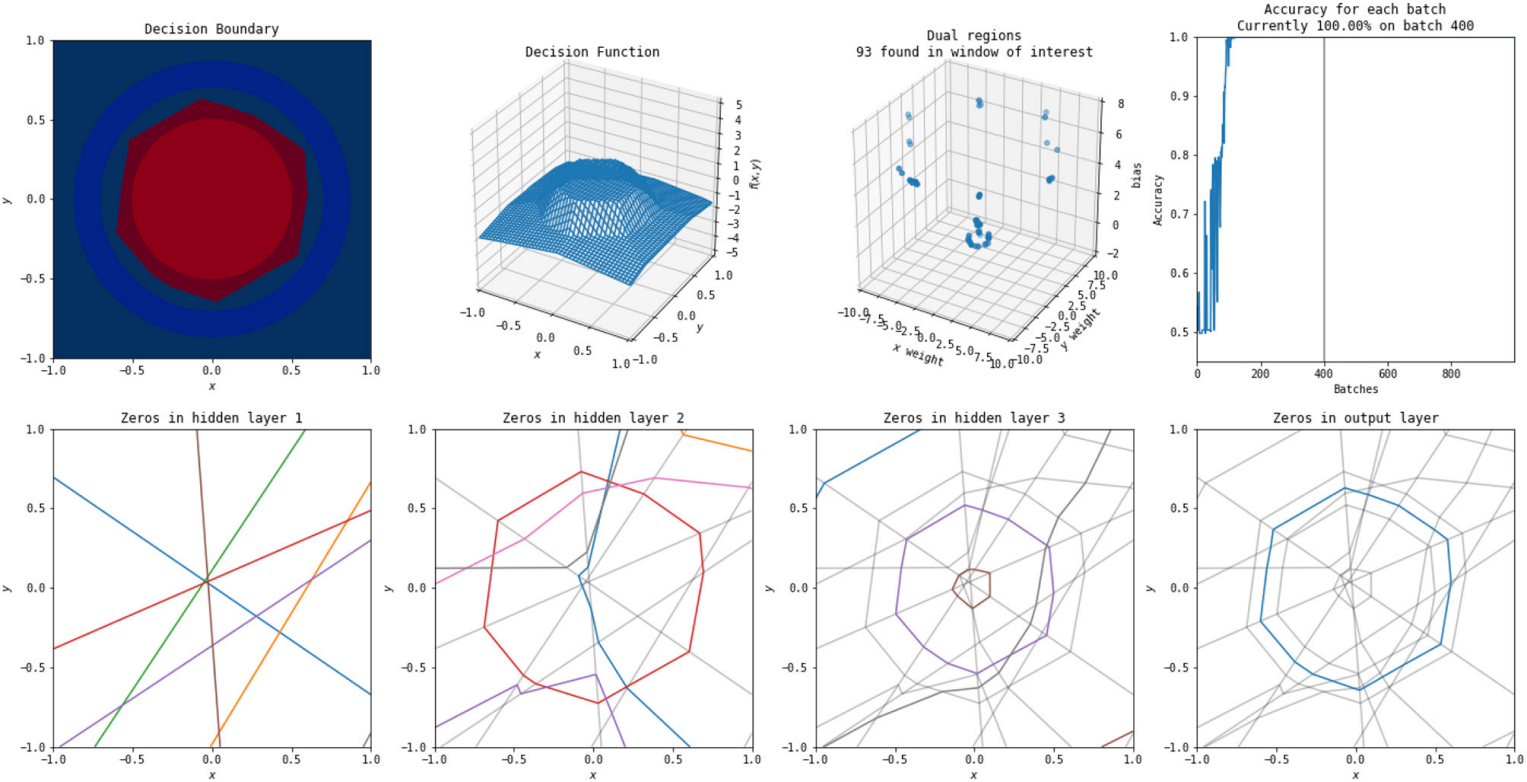
A solution to this problem found by a more complex network (three hidden layers, each with eight nodes). An animation of the training process of this network is available at https://www.youtube.com/watch?v=rANyD9t-X-c.

To illustrate how these polytopes and decision boundaries change as the neural network trains, we have two examples to compare as networks increase in complexity. One is the simplest possible network with three nodes in a single hidden layer, and the other is a far more complex network with three hidden layers each containing eight nodes. Still images of the polytope development throughout the training process for the simple network are shown in Figure 9 and the end result of the more complex network is shown in Figure 10. Full videos of the evolution of their polytope structure throughout the training process are available at https://www.youtube.com/watch?v=lpXQI-UJIZM and https://www.youtube.com/watch?v=rANyD9t-X-c, respectively.

As shown in the training animations, the increased complexity of the network in Figure 10 allows it to manipulate its non-linearities to create a closed region in fewer epochs than that of the simple network shown in Figure 9. This comes from the fact that the structure of the simple network forms a subnetwork of the more complex network. The more complex networks initialization is more likely to have activation boundaries in beneficial places for finding good solutions, as believed to occur with the lottery hypothesis (Frankle and Carbin, 2018). Additionally, the simple network does not find a solution every time using its training method, frequently finding locally optimal solutions that do not form closed regions and achieving only 50% accuracy.

**Figure 11:**
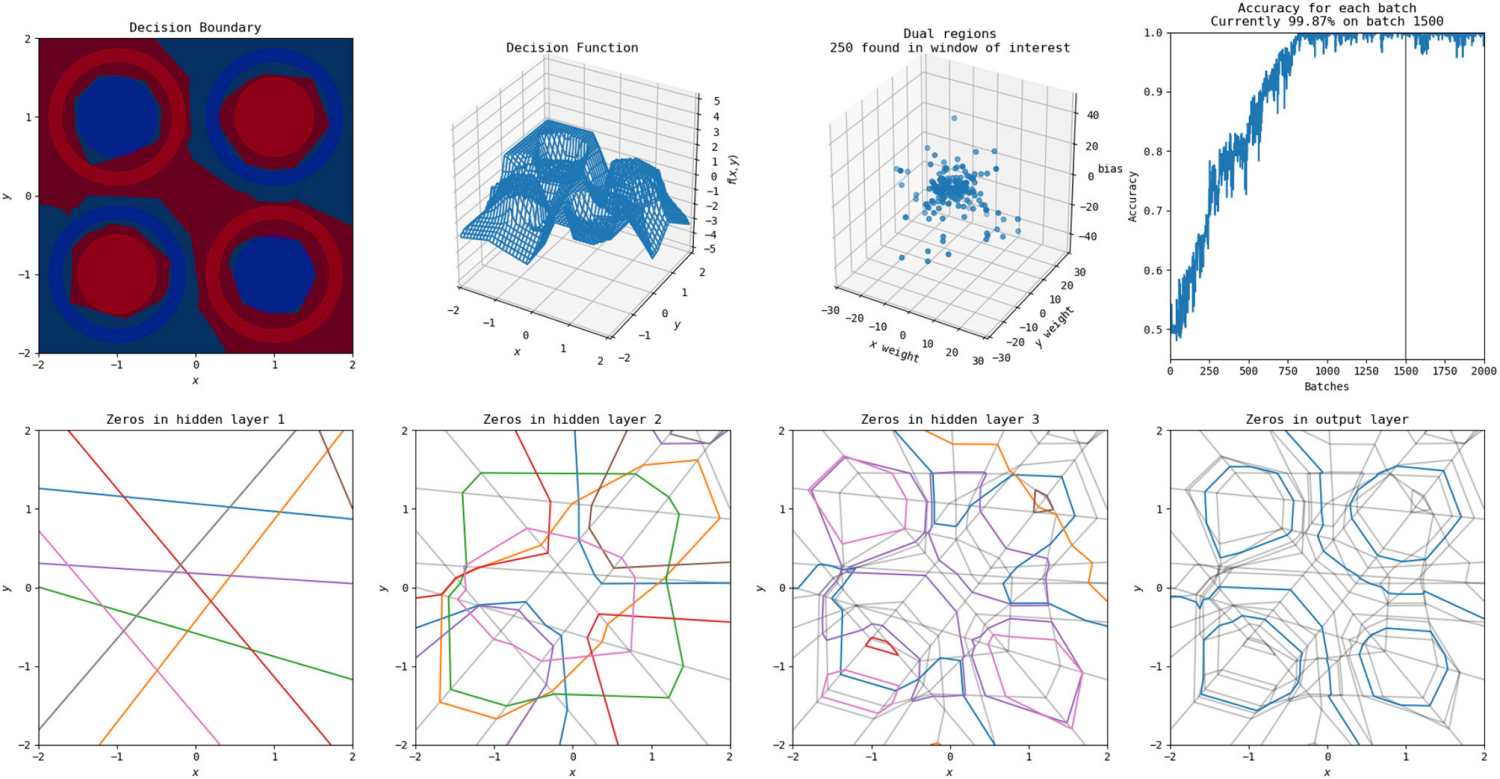
A solution to this problem found by a more complex network (three hidden layers, each with eight nodes). An animation of the training process of this network is available at https://www.youtube.com/watch?v=T_uoGBUOgUY.

An example of the polytopes constructed by a more complex network on combination of the XOR and circle vs. annulus problem is in Figure 11. A video of the training process is shown at https://www.youtube.com/watch?v=T_uoGBUOgUY. This network demonstrates a situation where the network requires a more complex structure to successfully classify. Additionally, the points in the dual do not cluster as they do in the original circle versus annulus problem.

It has previously been shown by Raghu et al. (2017) that earlier layers are more important than later layers for the quality of a network and certain visualizations of this were included in their study. These animations provide additional intuitive examples of this—the structures constructed by the early layers are passed on, and many of the deeper layers provide only slight modifications to the structures apparent in the first layers. Even in the more complex problem combining XOR and the circle versus annulus problem, the second layer forms the bulk of the structure for classification, with the third and final layers only refining it.

Using simple visualizations of this sort provide a holistic view of certain network behaviors. Intuitive understanding of existing hypotheses and theorems and inspiration for further investigation can be gained. In this case, the visualizations and animations demonstrate results such as the lottery hypothesis (Frankle and Carbin, 2018) and the influence of earlier layers (Raghu et al., 2017) visually to potentially enhance understanding of the phenomena. It also allows for identification of further areas of interest—in this case, the idea that networks trained on the same problem can exhibit significant similarity, and that there is possible pruning that can be done on networks that are more complex than necessary for representing a solution despite their increased complexity allowing a good solution to be found more effectively. Additionally, they show that the more complex network on the circle problem can have hidden layers removed, despite that method being relatively rare in studies of network pruning (Blalock et al., 2020).

### 3.2 Clustering sampled local linear functions

Using the methods discussed in Secton 2.4.1 and the networks described in Section 2.5, we can investigate the effect of clustering the linear weights for MNIST trained neural networks. Accuracies for this process with different numbers of cluster centers are shown in Table 1.

**Table 1:** The accuracies of networks on the MNIST dataset after applying *k*-means clustering to their collection of local linear maps.

**# Clusters**	**Dense**	**Conv1**	**Conv2**	**Inception**	**ResNet**
Original	9603	9807	9804	9908	9892
1	8679	6766	6366	974	1432
10	9231	8639	8672	9660	8166
100	9434	9382	9421	9689	8458
1000	9508	9586	9603	9695	8982
10000	9554	9696	9673	9752	9381

Values reported are the number of correctly labeled test set samples out of 10,000. Note that the number of clusters for a given network is technically 10 times larger than stated in the table—each of linear mappings corresponding to a digit is clustered separately.

Networks with less complex architectures capture the near-linear behavior of the MNIST dataset well. The dense, single-hidden-layer network, in particular, is able to recover a solution close to linear classifiers in the single cluster case, suggesting that the network replicates linear behavior well. This matches previous study that shows that single-hidden-layer wide networks tend to behave in highly linear ways.

Inception and ResNet, however, have near random performance in the single cluster case. This suggests that the networks are transforming the data in such a way that the transformation cannot be approximated linearly, which matches the complexity of architectural structure that those networks have. The basic convolutional networks perform poorly, but significantly better than random, suggesting that their transformation is non-linear but has some linear properties.

As the number of clusters increases, inception quickly recovers a high degree of accuracy, achieving better accuracy than the original dense network with as few as 10 clusters. This suggests that inception identifies a piecewise linear mapping from the input space that can be reasonably well approximated by as few as 10 regions. This means that inception has a high degree of redundancy when trained on MNIST, and could likely be pruned significantly while maintaining original accuracy. This also could be a sign that inception generalizes well on this dataset; it does not maintain a high level of complexity and uses relatively simple methods to classify the data.

This behavior is not matched by the basic convolutional networks or ResNet, with their performance remaining poor. The basic convolutional networks are able to recover for better accuracy than the original dense network with 10,000 clusters (1/6 of the number of training samples), but ResNet maintains poor accuracy throughout. This means that the polytope structure of ResNet cannot be simplified easily in this manner—either a more sophisticated method is necessary to identify ways of simplifying the polytope structure, or the network has a high degree of complexity that can not be reduced.

### 3.3 Affine maps between sampled local linear functions

**Figure 12:**
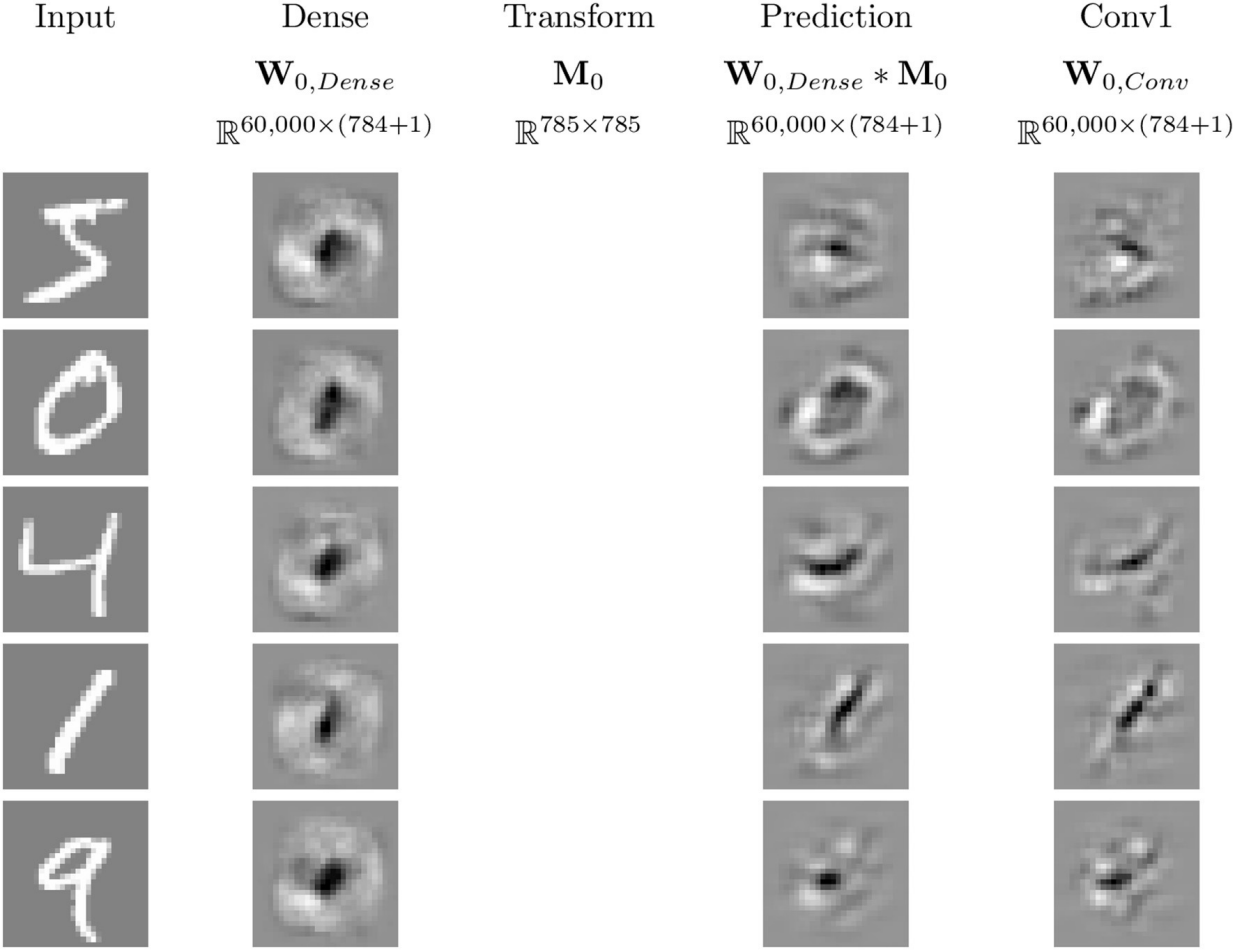
Example image representations of the affine mapping from the dense network to the simple convolutional network trained on MNIST. White pixels correspond to positive values, black pixels to negative values, and gray pixels to zero.

Using the methods discussed in Secton 2.4.2 and the networks described in Section 2.5, we can investigate the effect of transforming between the linear weights for different MNIST trained neural networks. Examples of this are illustrated for five input samples for **W**_0, *dense*_ and **W**_0, *conv*_ in Figure 12. Qualitatively, the mapped linear regions are similar, but not equivalent, to the target. One point to note is that all of the linear regions for the dense network appear qualitatively similar, with their shape matching that of a zero. The convolutional network does not follow this pattern, having different patterns for each of the input samples, and the transformed dense regions are able to replicate those patterns despite their visual similarities.

Table 2 shows the results of the affine mapping trained on the training set and evaluated on the testing set for the five networks trained on MNIST. No mapped network performs better than its original accuracy or the original accuracy of the network it is transformed to match. Despite this decrease in accuracy from the original networks, a high level of accuracy is maintained for many of the mappings. This is not necessarily unexpected, as all five networks are attempting to approximate the same function because they are trained to solve the same problem using the same loss function. However, accuracy does not necessarily tell the whole story. A high level of performance is preserved, but it may be the case that slight variations in accuracy represent significant, qualitatively meaningful differences in what the networks do. Even so, these results demonstrate that there is ostensibly an interesting relationship between these different networks and their similar behaviors.

**Table 2:** Number of correct labels on the test set (out of 10,000) after applying affine mappings to the linear mappings of MNIST trained neural networks.

		**To**				
		**Dense**	**Conv1**	**Conv2**	**Inception**	**ResNet**
**From**	Dense	9603	9536	9519	9290	9068
	Conv1	9567	9807	9776	9662	9588
	Conv2	9562	9786	9804	9644	9579
	Inception	8868	9488	9511	9908	9536
	ResNet	9320	9738	9739	9838	9892

Diagonal elements are the original accuracies of the networks.

The dense network and the basic convolutional networks are able to transform to each other reasonably well. Transforming to and from the dense network achieves accuracy near its original, and the basic convolutional networks with equivalent architectures and training methods are able to nearly replicate their original accuracies with transforming between each other. This suggests overlap in how the networks transform the data to achieve their networks, with the convolutional networks achieving additional complexity that allows them to increase their accuracy.

Interestingly, the inception network replicates the other networks poorly, with a significant drop in accuracy mapping to the dense network, accuracy near the dense networks transformation to the basic convolutional networks for them, and accuracy below the original dense network when transforming to ResNet. This matches the clustering results, where the network did not have a good single linear representation, explaining its lack of success mapping to the dense network, but recovers accuracy quickly with 10 clusters, suggesting a possible simple transformation that does not easily replicate the success of the other networks despite its success by itself.

ResNet, however, is able to replicate everything except the dense network well. It comes close to the original accuracies of the basic convolutional networks when mapping to them, as well as both its and inception's accuracies when mapping to inception. This suggests that the linear regions it uses contain the information that the other networks use for classification. Together with the results with clustering, this suggests that ResNet maintains a complex transformation from the input space.

The lack of symmetry between inception and ResNet is interesting. ResNet is able to approximate inception well, but inception is not able to approximate ResNet well. This means that there is a qualitative difference between the methods these networks are using for classification, despite their near equivalent original accuracies. This suggests, due to both of them achieving success, that ResNet identifies information that may not generalize, as inception is able to perform equivalently using a representation that appears simpler. Identifying both the overlap and differences in the methods these networks use for classification provides a way for identifying possible improvements for both networks.

By identifying the similarity between the linear regions of networks trained on the same or similar datasets, it is possible to gain a deeper understanding of the networks' behavior. Dissimilar representations suggest the exploitation of different information across the networks, meaning that the network behaviors can potentially be combined to improve effectiveness. The ability for one network to effectively recreate the representations of another suggests that the first network exploits the information the other contains meaning that differences in accuracy can come down to more effective exploitation, rather than identifying qualitative differences.

## 4 Discussion

Identifying patterns in simple neural networks trained on low-dimensional toy problems can provide meaningful insight and intuition for patterns that are replicated in modern neural networks trained on high-dimensional complex problems. These patterns can assist in gaining deeper insight into the behavior and in suggesting methodologies that can be applied to those complex networks. We have extended the work of Raghu et al. (2017) in visualizing the polytope structure of neural networks with two inputs by constructing animations of the evolution of the polytope structure. These animations demonstrate how early layers have significant influence over the structure of subsequent layers and how the polytope structures form through training.

Additionally, we have shown experimentally that even complex neural networks such as inception can have the complexity of their underlying polytope partitioning of the input space highly reduced. The linear regions of all networks considered, except ResNet, can be clustered to as few as 10 cluster centers for networks trained on MNIST while preserving much of their accuracy.

We have also shown experimentally that the linear regions of different networks are similar under an affine mapping. Applying such an affine mapping preserves a high level of accuracy in the resulting classifier, suggesting that many of the considered networks are solving problems in qualitatively similar ways. By comparing accuracies of mapped networks, we are able to determine where networks may have qualitatively dissimilar behavior in a way that suggests poor generalization or information that can be exploited to improve network behavior.

We provide support for the tantalizing idea that different networks converge to similar solutions that have a great deal more simplicity than would be suggested by their complex architectures. Further investigations of this area could allow for identification of patterns across disparate networks that allow for a more refined understanding of both training networks and modifying them to be effective in full usage. We would like to continue to explore the extent to which that idea is correct for modern neural networks through extensions to more complex datasets and network pruning methodologies.

Although MNIST provides high-dimensional image data, the dataset itself is relatively simple. Extending the similarity and clustering methods to more complex datasets would provide deeper insight into how complexity of dataset can influence the similarity and complexity of neural networks trained on them. Each of the networks trained here, despite their distinct architectures, used similar training methodologies. Extending the study by Zhang and Wu (2019) to investigate how different training methodologies and regularization techniques impact the similarity of network behavior would allow for an understanding of how those methods impact polytope structures.

Additionally, network pruning research provides a natural field where the ability to compare the similarity of two networks, linear regions would be useful. Identifying the degree to which a network can be simplified without impacting its ability to successfully classify can be difficult using only accuracy as a metric (Blalock et al., 2020). Comparing the behavior of those networks directly while being able to treat the interior of the network as a black box provides a promising technique for identifying success.

## Data availability statement

The datasets presented in this study can be found in online repositories. The names of the repository/repositories and accession number(s) can be found in the article/Supplementary material. Code accessible at https://github.com/bsattelb/local-linearity-of-relu-neural-networks/tree/master.

## Author contributions

BS: Conceptualization, Methodology, Software, Writing—original draft, Writing—review & editing. RC: Conceptualization, Writing—review & editing. MK: Conceptualization, Writing—review & editing. CP: Conceptualization, Writing—review & editing. RB: Conceptualization, Writing—original draft, Writing—review & editing.

## Supplementary material

Video 1.AVI An animation showing the training process of a simple neural network training to classify a points on a circle vs. points on a surrounding annulus. Polytope structure, local linear weights, decision boundary, and the output of the network are shown through the training process.

Video 2.AVI An animation showing the training process of a relatively complex neural network training to classify a points on a circle vs. points on a surrounding annulus. Polytope structure, local linear weights, decision boundary, and the output of the network are shown through the training process.

Video 3.AVI An animation showing the training process of a neural network training to classify points on four circles with classes matching the XOR problem and annuli surrounding each circle with opposite classification. Polytope structure, local linear weights, decision boundary, and the output of the network are shown through the training process.
